# Genetic mutations in lymphocytic variant of hypereosinophilic syndrome: study of five siblings

**DOI:** 10.3389/fmed.2025.1679484

**Published:** 2025-12-18

**Authors:** Molly Walkenhorst, Malay K. Basu, Wei Cui, Manish Kumar, Anusha Vallurupalli, Andrea Sitek, Xinyang Zhao, X. Long Zheng, Da Zhang

**Affiliations:** 1 Department of Pathology and Laboratory Medicine, The University of Kansas Medical Center, Kansas City, KS, United States; 2 Institute of Reproductive and Developmental Sciences, The University of Kansas Medical Center, Kansas City, KS, United States; 3 Department of Hematoloy and Medical Oncology, School of Medicine, Emory University, Atlanta, GA, United States; 4 Division of Allergy, Clinical Immunology, and Rheumatology, The University of Kansas Medical Center, Kansas City, KS, United States

**Keywords:** lymphocytic variant hypereosinophilic syndrome, clonal T-cells, eosinophilia, STAT3, immune dysregulation, transcriptional regulation

## Abstract

**Introduction:**

Lymphocytic variant hypereosinophilic syndrome (L-HES) is a rare subtype of hypereosinophilic syndrome driven by aberrant T-cell clones that promote eosinophilia through interleukin-5 (IL-5) overproduction. While clonal T-cell receptor (TCR) rearrangements are a hallmark, the underlying genetic landscape remains poorly defined.

**Methods:**

We report a familial case series involving five siblings, three symptomatic and two asymptomatic, with comprehensive clinical, immunophenotypic, and genomic evaluations. Whole-exome sequencing (WES) was performed to identify rare germline variants contributing to disease susceptibility. The index patient (EOS1) presented with clonal CD3^−^CD4^+^ T cells, marked eosinophilia, and papillary thyroid carcinoma (PTC). Flow cytometry, TCR gene rearrangement studies, and a targeted 141-gene NGS panel were conducted, followed by whole-exome variant calling and annotation.

**Results:**

EOS1 exhibited classic L-HES features and a positive TCR clonality test. Exome analysis revealed several nonsynonymous variants of uncertain significance in genes related to transcriptional regulation (ZNF257, MLLT1, BRD9), immune signaling (TESPA1, LRCH4, DHX58), and oncogenesis (CTAGE4, RGPD5). No STAT3 or recurrent mutations were identified. Several variants were shared among affected siblings but absent in unaffected controls, suggesting a possible hereditary predisposition.

**Discussion:**

This study highlights novel germline variants potentially associated with L-HES pathogenesis and expands the genomic spectrum beyond previously implicated somatic mutations. Our findings support the role of immune dysregulation and genetic predisposition in L-HES and underscore the importance of broader genomic profiling in familial cases. Functional validation and long-term monitoring are essential for risk stratification and early detection of malignant transformation.

## Introduction

Lymphocytic variant hypereosinophilic syndrome (L-HES) is a distinct subtype of hypereosinophilic syndrome (HES), characterized by the presence of an aberrant or clonal T-cell population that drives eosinophil proliferation through the overproduction of eosinophil-promoting cytokines, particularly interleukin-5 (IL-5). In contrast to myeloid HES, which arises from primary hematopoietic stem cell abnormalities, L-HES is primarily driven by immune dysregulation (1–3).

Patients with L-HES typically exhibit an immunophenotypically abnormal T-cell subset—most commonly CD3^−^CD4^+^ and, less frequently, CD3^+^CD4^+^—detectable by flow cytometry and molecular techniques (4). These aberrant T cells chronically stimulate IL-5 production, resulting in persistent eosinophilia and infiltration of eosinophils into target tissues, with the skin, lungs, and gastrointestinal tract most affected.

Recent molecular studies have shed light on the pathogenesis of L-HES. Although no consistent chromosomal translocations or driver mutations have been universally identified, various genetic abnormalities have been reported in subsets of patients. Clonal T-cell receptor (TCR) gene rearrangements, detected by polymerase chain reaction (PCR) or next-generation sequencing (NGS), are considered a hallmark of L-HES and indicate underlying T-cell clonality.

Furthermore, somatic mutations in genes involved in T-cell signaling and proliferation—such as STAT3 and STAT5B—have been implicated, particularly in cases progressing to T-cell malignancy. Gain-of-function mutations in STAT3, for example, have been linked to dysregulated cytokine signaling and enhanced IL-5 production, potentially worsening eosinophilia (5–7).

Epigenetic modifications, including hypermethylation of regulatory genes involved in T-cell differentiation, may also play a role in disease pathogenesis, although further investigation is needed to clarify their contribution.

Despite ongoing advances in understanding the immunologic and molecular basis of L-HES, several challenges remain. The mechanisms underlying aberrant T-cell expansion and potential transformation are not fully understood. Importantly, a subset of patients with L-HES may progress to T-cell lymphoma, emphasizing the need for long-term monitoring and early identification of malignant transformation.

In this study, we report a family of five siblings, three of whom are symptomatic and two asymptomatic. The asymptomatic siblings served as intra-familial controls to compare genetic alterations observed in the affected individuals.

## Materials and methods

This research study was reviewed and approved by the Institutional Review Board (IRB) at the University of Kansas Medical Center. Five siblings from the same family, all sharing the same biological parents, were included in the study. At the time of specimen collection, their clinical statuses were as follows: EOS1, a 16-year-old male, presented with the most severe symptoms; EOS2 (11-year-old male) and EOS5 (6-year-old male) had mild symptoms; EOS3 (8-year-old male) and EOS4 (12-year-old female) were asymptomatic. EOS1 exhibited skin eczema, absolute eosinophilia, an abnormal CD3^−^CD4^+^ clonal T-cell population, and papillary thyroid carcinoma (PTC). EOS2 and EOS5 presented with eczema and asthma and were managed symptomatically. However, due to insurance limitations and provider-related factors, these siblings were evaluated and treated at another institution; therefore, their CBC data, obtained externally, were not available for review at our center, and they did not undergo the same comprehensive evaluation as the index patient, EOS1. Whole peripheral blood specimens were collected from all five siblings, and genomic DNA was extracted using Qiagen DNA Extraction and Purification Kits (Qiagen, Germantown, MD, United States). Purified DNA was submitted to BGI (Cambridge, MA, United States) for whole exome sequencing.

### Alignment and variant calling

The pipeline for variant calling has been described earlier (8). Read quality was assessed using FastQC (version 0.11.5).^1^ To ensure high-quality data, raw sequencing reads were trimmed and filtered with Trimmomatic (version 0.36) (9) keeping the quality scores larger than 20. The processed reads were then aligned to the UCSC hg19 human reference genome using the BWA-MEM algorithm provided by BWA (version 0.7.10-r789).

Variant calling followed the best practices outlined by the Genome Analysis Toolkit (GATK; version 3.7.0).^2^ In summary, this involved: (1) marking duplicates with Picard’s MarkDuplicates tool (version 2.8.3); (2) performing local realignment around indels using GATK with RealignerTargetCreator and IndelRealigner; (3) conducting base quality score recalibration via GATK’s BaseRecalibrator; and (4) generating genomic VCF (GVCF) files using HaplotypeCaller, applying a minimum base quality threshold of 30.

The GVCF files from all samples were jointly genotyped using GATK’s GenotypeGVCF tool. Subsequent variant filtering was carried out using GATK’s Variant Quality Score Recalibration (VQSR), incorporating data from HapMap and the 1,000 Genomes Project. The final variant call format (VCF) files were annotated with ANNOVAR (10), incorporating information from CADD scores, ClinVar, dbNSFP, 1,000 Genomes allele frequencies, and snp138 databases. Common variants with an allele frequency above 0.05 in the 1,000 Genomes dataset were excluded to retain only rare variants for downstream analysis. An additional filter was applied to retain variants with a minimum read depth (DP) of 10.

## Results

### Clinical and laboratory findings in index case (EOS1)

Complete blood count (CBC) revealed hemoglobin 14.0 g/dL, hematocrit 41.9%, RBC 4.7 × 10^9^/L, MCV 88.7 fL, MCH 29.7 pg, MCHC 33.5 g/dL, RDW 14%, WBC 11.3 × 10^6^/L, and platelet count 321 × 10^6^/L. The differential count showed 30% segmented neutrophils and bands, 23% lymphocytes, 4% monocytes, and 42% eosinophils, with an absolute eosinophil count of 4.8 × 10^6^/L.

Bone marrow flow cytometry demonstrated eosinophilia and 3% atypical T-cells with an immunophenotype of CD3^−^, CD4^+^, CD2^+^, CD5^+^, CD7^−^, CD8^−^. Skin biopsy revealed atopic dermatitis with superimposed lichen simplex. Total thyroidectomy identified a 1.7 cm PTC with lymph node metastasis.

T-cell receptor (TCR) gene rearrangement testing was positive, consistent with clonal T-cell proliferation. Molecular studies for JAK2, CALR, and MPL were negative. Targeted next-generation sequencing (NGS) of 141 hematologic malignancy—associated genes (see Supplementary Table S1) identified two Tier 3 variants of uncertain clinical significance: MLH1 p.T347N and DNMT3A p.V372I.

By comparing the exonic genetic variants of the index patient (EOS1) and two symptomatic siblings (EOS2, EOS5) with those of two unaffected siblings (EOS3, EOS4), we identified variants present in all three symptomatic individuals but absent in the unaffected siblings, as summarized in Table 1.

**Table 1:** Genetic variants summary.

Chr	Location	Ref	Alt	Affected region	Gene	Mutation type	SnpID	1K Geome freq	SIFT prediction	PolyPhen-2 prediction	AlphaMissense score	Gnomad freq
chr10	123844172	G	T	Exonic	TACC2	Nonsynonymous SNV	rs41288002	0.0171725	D	B	0.243	0.0357
chr10	126678196	C	T	Exonic	CTBP2	Nonsynonymous SNV	nan	—	D	D	0.795	0.0002
chr11	1018459	G	T	Exonic	MUC6	Nonsynonymous SNV	rs199760270	—	T	P	0.068	4.594 × 10^−5^
chr11	1018473	A	T	Exonic	MUC6	Nonsynonymous SNV	rs112923701	—	D	D	0.091	8.576 × 10^−5^
chr11	124267148	C	T	Exonic	OR8B3	Nonsynonymous SNV	rs653057	—	T	B	0.063	0.2121
chr1	152188847	A	G	Exonic	HRNR	Nonsynonymous SNV	rs145667921	—	T	B	0.076	0.0840
chr1	248437002	C	T	Exonic	OR2T33	Nonsynonymous SNV	rs146010336	—	T	P	0.156	0.0137
chr12	55368291	C	T	Exonic	TESPA1	Nonsynonymous SNV	rs62623446	0.0231629	D	D	0.097	0.0446
chr16	70211199	T	C	Exonic	CLEC18C	Nonsynonymous SNV	rs62052576	—	T	B	0.095	0.0348
chr17	32957114	G	A	Exonic	TMEM132E	Nonsynonymous SNV	rs56879769	0.038738	T	B	0.081	0.0268
chr17	40263458	T	C	Exonic	DHX58	Nonsynonymous SNV	rs34891485	0.0159744	T	B	0.059	0.0362
chr18	11610583	G	T	Exonic	SLC35G4	Nonsynonymous SNV	rs112309017	—	—	—	0.101	0.0490
chr18	11610595	G	A	Exonic	SLC35G4	Nonsynonymous SNV	rs200189946	—	—	—	0.087	0.0550
chr19	22270899	G	T	Exonic	ZNF257	Nonsynonymous SNV	nan	—	—	B	0.091	—
chr19	22270928	T	C	Exonic	ZNF257	Nonsynonymous SNV	nan	—	—	B	0.145	—
chr19	22270929	G	A	Exonic	ZNF257	Nonsynonymous SNV	nan	—	—	B	0.122	—
chr19	22270934	G	C	Exonic	ZNF257	Nonsynonymous SNV	nan	—	—	B	0.134	—
chr19	22270935	G	A	Exonic	ZNF257	Nonsynonymous SNV	nan	—	—	B	0.094	—
chr19	6222353	C	T	Exonic	MLLT1	Nonsynonymous SNV	rs11880101	0.0133786	T	B	0.065	0.0294
chr2	113127775	G	C	Exonic	RGPD5, RGPD8	Nonsynonymous SNV	rs15849	—	T	B	0.058	0.0151
chr2	28827625	C	T	Exonic	PLB1	Nonsynonymous SNV	rs34289907	0.043131	T	D	0.115	0.0729
chr2	96617138	T	C	Exonic	ANKRD36C	Nonsynonymous SNV	nan	—	T	—	0.173	0
chr4	190874234	C	T	Exonic	FRG1	Nonsynonymous SNV	rs200620299	—	D	D	0.989	0.4250
chr5	878525	T	A	Exonic	BRD9	Nonsynonymous SNV	rs508016	—	T	B	0.132	0.3191
chr7	100175010	C	T	Exonic	LRCH4	Nonsynonymous SNV	rs140761835	0.00499201	T	P	0.075	0.0124
chr7	143882673	C	T	Exonic	CTAGE4	Nonsynonymous SNV	rs1635873	—	T	B	0.049	0.2636
chr7	99159522	C	G	Exonic	ZNF655	Nonsynonymous SNV	rs141089610	0.00758786	T	B	0.07	0.0147

Summary of important nonsynonymous single nucleotide variants (SNVs) detected across multiple chromosomal regions. The table lists chromosomal location (Chr), reference (Ref) and alternate (Alt) alleles, affected region, corresponding gene, mutation type, single nucleotide polymorphism identifier (SnpID), population allele frequencies from the 1,000 Genomes Project and gnomAD, and *in silico* predictions from SIFT, PolyPhen-2, and AlphaMissense. SIFT indicates functional impact as deleterious (D) or tolerated (T), while PolyPhen-2 predictions classify variants as benign (B), possibly damaging (P), or probably damaging (D). Higher AlphaMissense scores denote increased predicted pathogenicity.

## Discussion

Our index patient presented with classic features of L-HES, including peripheral eosinophilia and a clonal CD3^−^CD4^+^ T-cell population. In L-HES, eosinophilia is considered a reactive phenomenon secondary to the overproduction of eosinophilopoietic cytokines such as interleukin-5 (IL-5) and/or IL-3 by clonal T-cells, rather than a primary myeloid process (6). In this study, we identified a panel of nonsynonymous exonic variants. Although the clinical significance of these mutations remains uncertain, many affected genes are involved in transcriptional regulation, immune function, or oncogenesis, suggesting they may contribute to disease susceptibility, modulation, or progression.

### Transcriptional regulation and chromatin remodeling

Several mutations were identified in genes related to transcriptional control and chromatin remodeling. These include ZNF257 and ZNF655, both zinc finger proteins predicted to function as transcriptional regulators. ZNF655 has been associated with tumor progression in solid malignancies, while ZNF257 was among the top differentially expressed genes in a recent codon usage bias (CUB) analysis of severe combined immunodeficiency (SCID)-associated genes (11). That study emphasized the impact of codon bias and regulatory sequences on gene expression and proposed ZNF257 as a potential target for gene therapy in immune dysfunction. The recurrence of a ZNF257 mutation in our case may suggest a regulatory hotspot or dosage sensitivity, raising the possibility of a link between transcriptional dysregulation and immune abnormalities.

Mutations were also detected in MLLT1 and BRD9, both involved in chromatin remodeling. MLLT1 is a known fusion partner in MLL-rearranged leukemias (12); although no fusion was identified here, its mutation may signal an underlying predisposition to hematologic neoplasia. BRD9, a component of the non-canonical BAF (ncBAF) chromatin remodeling complex, is essential for normal hematopoiesis. BRD9 depletion alters transcriptional programs and induces apoptosis in hematologic malignancies (13).

Additional variants were found in TACC2, a centrosomal protein important for mitotic spindle formation, and FRG1, a gene implicated in muscle and vascular development as well as prostate cancer (14, 15). These may reflect broader disruptions in transcriptional or post-transcriptional regulation.

### Immune regulation and signal transduction

Multiple variants were found in genes with immune-regulatory roles, supporting the concept that eosinophilia in L-HES may result from immune dysregulation. ANKRD36C has been implicated in immune-mediated thrombotic thrombocytopenic purpura (9), while TESPA1 plays a crucial role in T-cell receptor (TCR) signaling and thymocyte development. TESPA1 dysfunction may lead to abnormal T-cell activation and increased cytokine production such as IL-5, a key driver of eosinophil proliferation (14).

LRCH4, a regulator of Toll-like receptor signaling, is increasingly recognized as an immune modulator (16). DHX58, a cytoplasmic helicase involved in RIG-I-like receptor signaling, may affect antiviral and proinflammatory responses (17, 18). Mutations in these genes may disturb normal cytokine signaling pathways, contributing to clonal T-cell expansion and eosinophilia in L-HES.

CLEC18C, a C-type lectin domain-containing protein, is believed to participate in innate immune recognition and metabolic regulation (19). A mutation in HRNR, a gene involved in skin barrier function, may be clinically relevant given the patient’s history of eczema. HRNR mutations have been associated with atopic dermatitis and other inflammatory dermatoses (20).

Variants were also detected in RGPD5 and RGPD8, both members of a gene family involved in nucleocytoplasmic transport and potentially in post-translational protein modification (21). Although their roles in immune function are unclear, disruption of these processes could have downstream effects on protein trafficking and immune signaling.

Interestingly, mutations were also observed in SLC35G4, a predicted nucleotide sugar transporter, and TMEM132E, a transmembrane protein associated with nonsyndromic hearing loss (22). Although these genes are not classically immune-related, other solute carrier family members (e.g., SLC26A4) have been linked to airway inflammation and hyperresponsiveness in asthma (23) and familial thyroid carcinoma (24), suggesting potential relevance in eosinophilic disorders and early on set thyroid carcinoma.

### Oncogenesis and tumor-associated antigens

In addition to genes involved in immune and transcriptional regulation, mutations were identified in oncogenesis-related genes. CTAGE4, initially described as a tumor-associated antigen in cutaneous T-cell lymphoma, may be significant in the context of clonal T-cell proliferation. Its expression in both epithelial and lymphoid tissues, along with its immunogenic potential, suggests utility as a biomarker or immune-modulatory target (12).

Mutations in OR8B3, OR2T33, and MUC6 were also detected. Although these genes are best known for roles in olfaction and mucosal function, olfactory receptors have been increasingly recognized for their activity in non-sensory tissues, including the immune system and epithelium (25). The functional significance of these variants remains unclear but may reflect immune-epithelial interactions or tissue-specific gene expression changes.

Given the pediatric onset of PTC in EOS1, an assessment of genes associated with PTC was of particular interest. A KRAS mutation in the 3′ untranslated region was identified in EOS1, EOS2, and EOS3. Point mutations in RAS genes (HRAS, NRAS, KRAS) have been reported in up to 25% of PTC cases, though their frequency is lower in pediatric populations (less than 10%) (26–28). BRAF mutations are also relevant in PTC. While the classic BRAF V600E mutation is the most common alteration in adult-onset PTC, it occurs less frequently in pediatric patients and is associated with a worse prognosis and treatment response. BRAF V600E is more prevalent in adolescents (ages 16–20), whereas BRAF fusions are more commonly seen in children under 10 years of age (26–28). In this case, no BRAF point mutation was detected, and due to limitations of the sequencing methods used, gene fusions could not be assessed. Notably, BRAF and RAS mutations in PTC are typically mutually exclusive (27). RET fusions, which are more common in younger pediatric patients, also could not be evaluated in this study (26, 28).

### STAT3 and broader implications

The STAT3 mutation previously described in L-HES (5) was not detected in our case. Similarly, a previous study involving seven L-HES cases reported no somatic mutations—including STAT3—in three patients who underwent next-generation sequencing using 28- and 81-gene panels (4). These findings may reflect limitations of targeted gene panels or the diverse mutational landscape of L-HES.

In summary, while the pathogenicity of many identified variants remains uncertain, their enrichment in genes related to transcriptional regulation, immune signaling, and oncogenesis underscores the complexity of L-HES and the potential contribution of genetic predisposition. The detection of multiple variants of uncertain significance (VUS) highlights the need for functional studies, family segregation analysis, and continued clinical monitoring. Broader genomic profiling may help clarify the molecular basis of L-HES and inform individualized approaches to diagnosis and management.

## Data Availability

The raw data supporting the conclusions of this article will be made available, on reasonable request to the corresponding author.

## Footnotes

1
www.bioinformatics.babraham.ac.uk/projects/fastqc/

2
http://gatk.broadinstitute.org

## Supplementary material

SUPPLEMENTARY FIGURE 1 Genetype heatmap. Heatmap showing the relationship between samples. A 0/1/2 table showing the SNV status was created from all samples using samtools: 0, 1, 2 represents absent, heterogygous and homozygous status of a particular SNV. The heatmap show only randomly sampled 500 variants.

SUPPLEMENTARY TABLE S1 Comprehensive annotation of eosinophilic mutation dataset. This dataset provides a comprehensive functional and population-level characterization of eosinophilic variant profiles. It includes detailed annotations of single nucleotide variants (SNVs) and small insertions/deletions (indels) identified across eosinophilic samples (EOS1–EOS5). Each row represents a unique genomic variant with positional, functional, and predictive annotations. Columns include sample-level mutation status, chromosomal coordinates (Chr, Start, End), reference (Ref) and alternate (Alt) alleles, and gene-based annotations from RefGene (Func.refGene, Gene.refGene, GeneDetail.refGene, ExonicFunc.refGene, AAChange.refGene). Clinical significance and database cross-references (CLINSIG, CLNDBN, CLNACC, CLNDSDB, CLNDSDBID) are provided where available. Functional predictions include SIFT, PolyPhen-2 (HDIV and HVAR), LRT, MutationTaster, MutationAssessor, FATHMM, PROVEAN, VEST3, CADD, DANN, fathmm-MKL, MetaSVM, and MetaLR, along with conservation metrics (GERP++, phyloP, phastCons, and SiPhy). AlphaMissense scores and rankings indicate predicted deleteriousness. Population allele frequencies from gnomAD [overall and by subpopulation: African (AF_afr), South Asian (AF_sas), American (AF_amr), East Asian (AF_eas), non-Finnish European (AF_nfe), Finnish (AF_fin), Ashkenazi Jewish (AF_asj), and other populations (AF_oth)] are included, along with total and sex-specific allele frequencies (AF_raw, AF_male, AF_female). Detailed description of each column is provided in a separate sheet in the file.
