# Supplementary figures and images for: Genetic mutations in lymphocytic variant of hypereosinophilic syndrome: study of five siblings
Source: Front Med (Lausanne). 2025 Dec 18;12:1679484. doi: 10.3389/fmed.2025.1679484 (PMC12756459; doi:10.3389/fmed.2025.1679484)

Genotype Heatmap (subset)

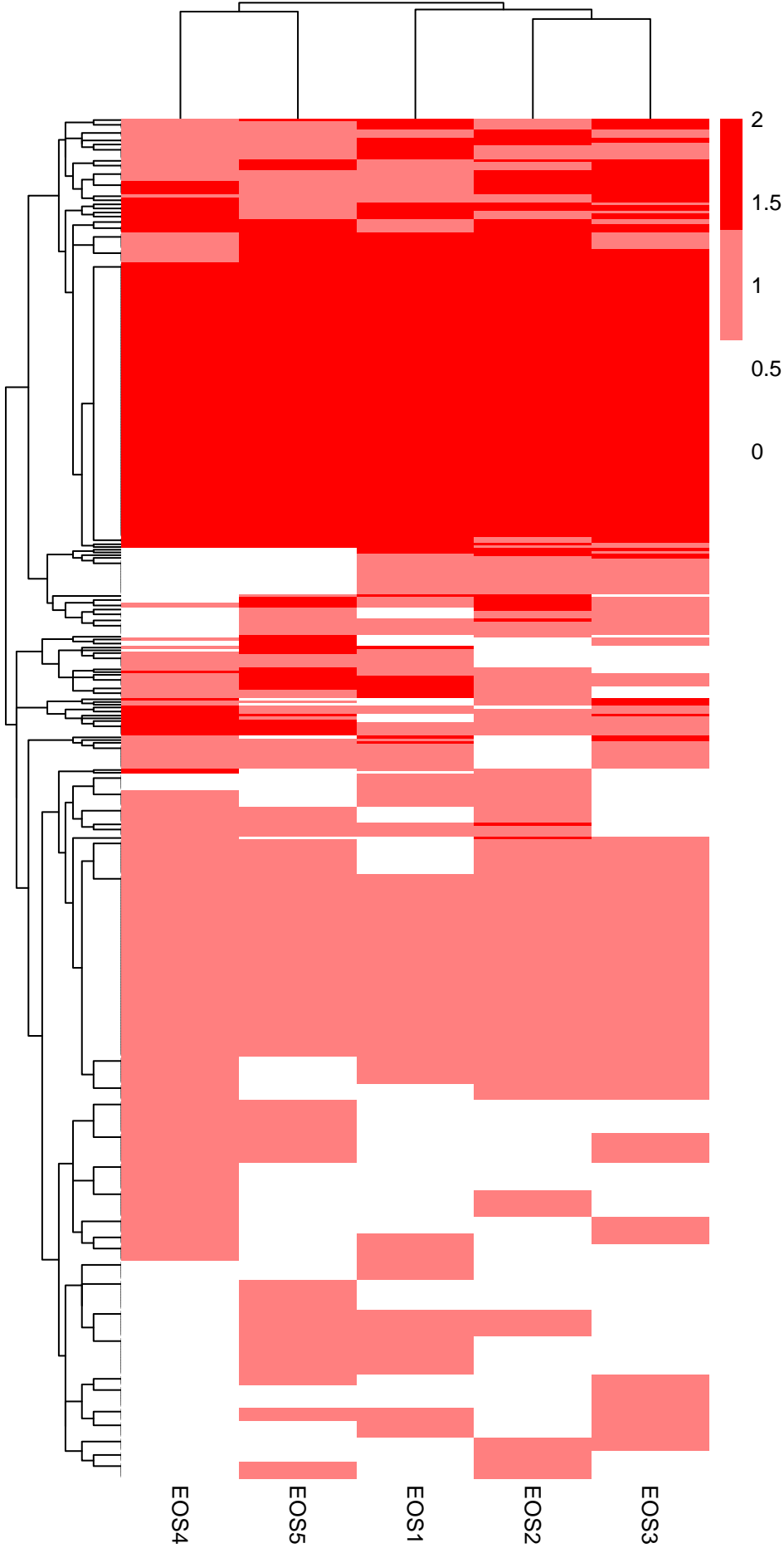

Supplement: Supplementary file 1 [file Image_1.pdf]
